# Tolerance to multiple climate stressors: a case study of Douglas‐fir drought and cold hardiness

**DOI:** 10.1002/ece3.2007

**Published:** 2016-02-26

**Authors:** Sheel Bansal, Constance A. Harrington, John Bradley St. Clair

**Affiliations:** ^1^ Pacific Northwest Research StationUSDA‐Forest Service3625 93^rd^ Avenue SWOlympiaWashington98512; ^2^ Pacific Northwest Research StationUSDA‐Forest Service3200 SW Jefferson WayCorvallisOregon97331

**Keywords:** Abiotic stress, climate change, common garden, genetic variation, Pacific Northwest, precipitation gradient, principal components analysis, *Pseudotsuga menziesii*, temperature gradient

## Abstract

1. Drought and freeze events are two of the most common forms of climate extremes which result in tree damage or death, and the frequency and intensity of both stressors may increase with climate change. Few studies have examined natural covariation in stress tolerance traits to cope with multiple stressors among wild plant populations.
2. We assessed the capacity of coastal Douglas‐fir (*Pseudotsuga menziesii* var. *menziesii*), an ecologically and economically important species in the northwestern USA, to tolerate both drought and cold stress on 35 populations grown in common gardens. We used principal components analysis to combine drought and cold hardiness trait data into generalized stress hardiness traits to model geographic variation in hardiness as a function of climate across the Douglas‐fir range.
3. Drought and cold hardiness converged among populations along winter temperature gradients and diverged along summer precipitation gradients. Populations originating in regions with cold winters had relatively high tolerance to both drought and cold stress, which is likely due to overlapping adaptations for coping with winter desiccation. Populations from regions with dry summers had increased drought hardiness but reduced cold hardiness, suggesting a trade‐off in tolerance mechanisms.
4. Our findings highlight the necessity to look beyond bivariate trait–climate relationships and instead consider multiple traits and climate variables to effectively model and manage for the impacts of climate change on widespread species.

## Introduction

Climate change is inherently a multivariate process, that is, many components of climate are changing simultaneously and unevenly. Most research on climate change has focused on the univariate effect of increased average temperatures, but changes in precipitation regimes and in extreme weather events are also co‐occurring (I.P.C.C. 2013). These multivariate changes in climate are occurring worldwide (Easterling et al. 2000; Guirguis et al. 2011). A recent example of multivariate climate change is occurring in California, USA, which has had extremely low precipitation combined with high temperatures, leading to its worst drought in 1200 years (Worrall 1971; Oribe and Kubo 1997). In the same years, much of the USA (including California) experienced a suite of record‐breaking cold temperatures, frost events, and snowfall. Multiple climate stressors, particularly with regard to summer and winter conditions, are likely to become more frequent in the future (Drew and Downes 2009; Bahn et al. 2014), which will undoubtedly impact the performance and persistence of many species (VanDerWal et al. 2013; Frank et al. 2015; Ma et al. 2015; Renwick and Rocca 2015).

Suitably, numerous overlapping mechanisms for coping with groups of related stressors, such as with flooding, soil compaction, and oxygen deficiency, or with drought, salinity, and cold, are encoded into the plant genome (Shinozaki and Yamaguchi‐Shinozaki 1996; Kasuga et al. 1999). Stress tolerance genes have evolved through generations of natural selection in response to extremes in temperature and precipitation (Van Mantgem and Stephenson 2007; Jackson et al. 2009). Selection of genes to tolerate one stressor often affects tolerance to other stressors (e.g., through pleiotropy, linkages, or correlated selection), resulting in genetic covariation in tolerances to multiple stressors among intraspecific populations (McKay et al. 2003; Hellmann and Pineda‐Krch 2007). Biophysical constraints (e.g., xylem structure) are also important with regard to tolerance to multiple stressors (Tyree and Cochard 1996). In some cases, acquiring greater tolerance to one stressor may reduce tolerance to another stressor (Laanisto and Niinemets 2015). Understanding these trade‐offs (or lack thereof) among traits is key for predicting species responses to global change factors, such as climate change.

Species with distributions that span large multivariate climate space are likely to have highly differentiated populations with respect to stress tolerance (Campbell and Sorensen 1973). Populations from areas that experience multiple climatic stressors, such as dry summers *and* cold winters, are likely to be tolerant to both drought and cold (Laanisto and Niinemets 2015). Coastal Douglas‐fir (*Pseudotsuga menziesii* var. *menziesii*) is an ecologically and economically important species that is widely distributed along the Pacific Coast of North America. This species is an excellent case study for understanding the geographic distribution of tolerance to both drought and cold stress because its range spans a large gradient in winter and summer conditions and the species exhibits strong genetic clines in tolerance to both of these stressors (St. Clair 2006; Eilmann et al. 2013; Bansal et al. 2015a,b).

As drought has been persistent for 4+ years throughout much of Douglas‐fir's range, particularly at the southern, warm edge of its distribution in California, tolerance to drought is of paramount concern for forest ecologists, land managers, and conservationists alike (Van Mantgem and Stephenson 2007). At the same time, extreme frost events have severely impacted forest health and productivity in recent and past decades (Duffield 1956; Peeler and DeBell 1987), and are expected to continue even with climate warming (I.P.C.C. 2013). Currently, Douglas‐fir is a candidate species for transferring populations from warmer to cooler locations (referred to as assisted migration) as a planned response to minimize negative effects of future warming on plant growth and survival (Rehfeldt et al. 2014). However, such population movements may lead to higher risk of frost damage in the short term because of maladaptation. Nevertheless, historical climate has undergone periods of extreme cold (e.g., glaciations) and extreme drought over evolutionary time scales (Worrall 1971), meaning that Douglas‐fir may have underlying genetic adaptations that remain unexpressed until extreme climatic events induce stress response mechanisms. Knowing the extent and geographic distribution of these “hidden” safety mechanisms will be essential for avoiding large‐scale mortality from assisted migration of Douglas‐fir.

**Figure 1:**
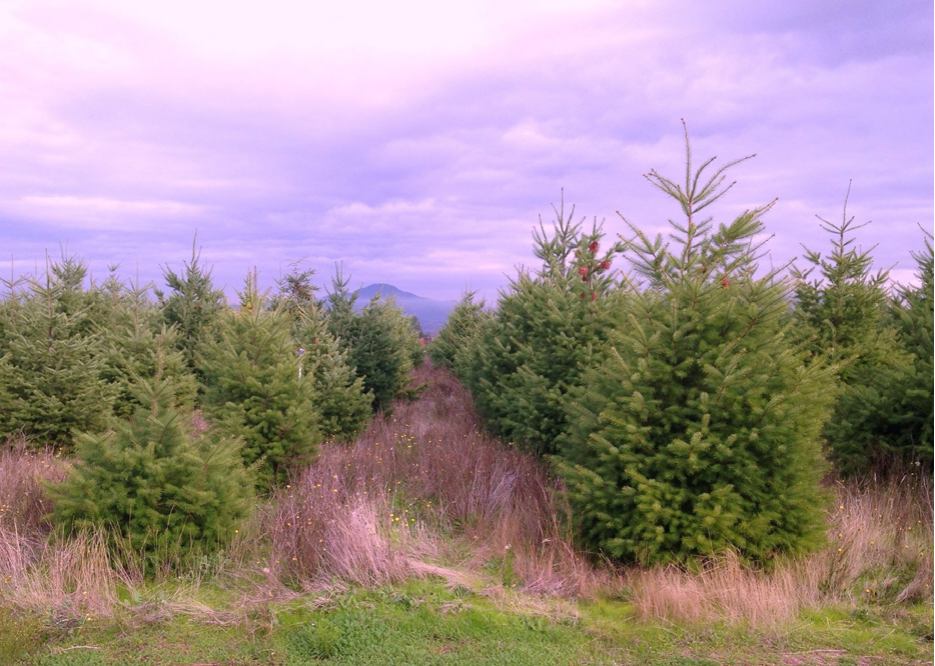
Coastal Douglas‐fir (*Pseudotsuga menziesii* var. *menziesii*) trees growing in an warm and arid common garden. Seed sources for trees on the left and right are from the California Sierra Nevada Mountains and Washington coast, respectively.

Common gardens are typically used to identify genetic variation among populations in their abilities to cope with stress, measured as differences in the expression of physiological/morphological traits associated with stress tolerance (i.e., genetic effects). The expression of stress tolerance traits is also responsive to the environmental conditions of the common garden (i.e., environmental effects). In addition, the environmental response may vary among populations, resulting in genetic × environment interactions (Greer and Warrington 1982; Jermstad et al. 2003). Multiple common gardens studies are able to quantify the relative influences of genetics, environment, and their interactions on the expression of stress tolerance traits. In two previous studies that used multiple common gardens, we separately assessed the relative influence of genetics, environment, and their interactions for drought hardiness (Bansal et al. 2015a) and cold hardiness (Bansal et al. 2015b) of Douglas‐fir. When wild populations of Douglas‐fir experience environmental stress under natural conditions, then genetics, environment, and the interactions all influence the observed *in situ* expressions of stress tolerance traits, which are the ultimate drivers of persistence through environmental stress. In this study, we explored how drought hardiness and cold hardiness covary among populations under more natural condition (i.e., when genetics, environment, and their interactions all influence trait expression). Therefore, we conducted new analyses of data from the two previous studies in which we combined drought hardiness traits measured in an arid common garden and cold hardiness traits measured in a cool garden on the same genotypes (Fig. 1). We used principal components analyses to combine drought and cold hardiness trait data into generalized “stress hardiness” traits to model geographic variation of stress hardiness across the Douglas‐fir range. This study will increase our understanding of how multiple climate variables have influenced the development and expression of tolerance to both drought and cold stress, and aid in developing strategies for assisted migration.

## Materials and Methods

A series of nine common gardens were established as part of a coastal Douglas‐fir reciprocal transplant study (called the Douglas‐fir Seed‐Source Movement Trial, SSMT) in the Pacific Northwest region of USA in Washington, Oregon, and northern California. Each common garden consisted of populations of coastal Douglas‐fir grown from seeds that were collected from regions throughout its native range in Washington, Oregon, and California. Seed‐source climates covered a range of dry/wet summers and cold/warm winters. Winter temperatures and summer precipitations were uncorrelated among seed‐source climates (Methods S1). In each region, five populations separated by at least 20 km were collected. Seeds for each population were collected from two open‐pollinated parent trees separated by at least 100 m. In fall 2008, two‐year‐old container‐grown seedlings were planted in a complete block design with families from the same region planted in randomly assigned locations in plots within each of four blocks at each garden. Trees were arranged in four rows with five trees per row at a 2.7 m square spacing within each plot (Methods S2 for experimental design).

Drought hardiness and cold hardiness were assessed in related studies in 2012 at three of the common gardens (cool, moderate, and warm) on 35 populations (Bansal et al. 2015a,b). For the current analyses, data from the cool and warm gardens were used because they represented the extremes with regard to annual temperature and precipitation. From the warm and arid garden, minimum transpiration (transpiration_min_, rate of water loss through the cuticle after stomatal closure), specific leaf area (SLA, amount of leaf area for water loss relative to leaf mass), and water saturation deficit (WSD, tissue water content relative to its maximum potential water content) were measured on branch samples collected at midday in midsummer during maximum moisture stress as indicators of drought hardiness (Bansal et al. 2015a). Transpiration_min_, water deficit, and SLA are parameters of plant water status that are intrinsically related to water loss, water conservation, and stress tolerance, respectively (Muller et al. 2011), can be quickly collected for many individuals, and are a consequence of morphological development and physiological adjustments integrated over time (Barrs 1968). For transpiration_min_ and SLA, higher values indicated faster rates of water loss and more leaf surface area for water loss per unit of mass, respectively, and thus lower drought hardiness. SLA can be influenced by environmental variables other than moisture status (e.g., light levels, soil nutrients, temperature) (Bansal and Germino 2008), which were controlled for in the experimental design to avoid confounding effects on SLA. For WSD, woody plants adapted to arid conditions have greater cell wall elasticity, which allows for more cellular water loss while avoiding membrane collapse (Lambers et al. 1998), and therefore higher values indicate greater drought hardiness.

We assessed transpiration_min_ as the rate of water loss under conditions of maximum stomatal closure through measurements of leaf‐drying curves from a clipped twigs 8 cm in length (Hygen 1951; Cape and Percy 1996; Anfodillo et al. 2002). The base of each clipped twig was sealed with warmed grafting wax. Twigs were initially weighed and then reweighed every 24 h for 7 days. As the twigs dried, the slope of the linear portion of the leaf‐drying curve was considered the rate of transpiration_min_ (also referred to in the literature as “minimum conductance”) and is associated with stomatal closure (Marks and Lechowicz 2007) and the turgor loss point (Burghardt and Riederer 2003). For SLA, a sample of 8 fresh needles was removed from fresh twigs to determine fresh leaf area using image processing software, dried, and then weighed (±0.1 mg). Specific leaf area was calculated as fresh leaf area (cm^2^) divided by dry leaf mass (g). For WSD, a second twig was initially weighed after collection (fresh weight) and then placed into a sealed plastic bag with 2 ml of distilled water added. Twigs were angled such that the base of each twig was submerged in water. Following complete hydration, twigs were removed from bags and immediately weighed (turgid weight). Twigs were then dried at 65°C for 72 h and then reweighed (dry weight). WSD was calculated as (turgid weight–fresh weight) / (turgid weight–dry weight) × 100 (Barrs 1968).

From the cool garden, cold hardiness was measured on branch samples from the same seed‐sources at the end of October 2012 following the period of cold acclimation. Bud, stem, and needle damage were assessed following artificial freeze tests (Bansal et al. 2015b), with lower cold damage corresponding to greater cold hardiness. Four test temperatures were used to produce a range of damage scores. Branch samples were grouped by block wrapped into packets of moist cheesecloth, and covered with aluminum foil. Sample packets were placed in a programmable freezer (Forma Scientific Model 8270/859M, Thermo Fischer Scientific Inc., Waltham, MA, USA) with a built‐in temperature controller (model WestM3750) overnight at −2°C to equilibrate them. The following day, freezer temperatures were lowered by 1–3°C per hour until the warmest test temperature was reached. The temperature was maintained for 1 h and then one packet from each test site was removed from the freezer. The process of reducing temperature by 1–3°C, maintaining for 1 h, and then removing packets continued for the second, third, and fourth test temperatures. Immediately upon removal from the freezer, the packets were placed in a 4°C refrigerator overnight to allow samples to slowly thaw. The packets were then kept at room temperature for 6–7 days to allow symptoms of cold damage to develop. The freeze‐treated samples were visually scored for cold damage as the percentage of each type of tissue (bud, needle, and stem) showing injury (yellowing or browning). Stems and buds were tangentially cut to assess percent damage. By taking the mean damage score of the four test temperatures, we increased the precision of our cold hardiness assessment for each population (Aitken and Adams 1996, 1997; Anekonda et al. 2000).

### Data analyses

The primary purpose of this analysis was to understand the overall pattern of covariation in tolerance to stress, as opposed to individual traits associated with drought or cold hardiness (which are covered in detail in Bansal et al. 2015a,b). We used trait values for drought and cold hardiness from the warm and cool gardens, respectively, because innate variation in tolerances to drought and cold among populations is most strongly expressed in response to the dry and cool climates of those respective gardens. In addition, using stressful gardens accounted for environmental and genetic × environment interactions; thus, the expression of traits is more representative of actual expression under natural conditions. We used principal components analysis (PCA) to combine data on drought and cold hardiness traits together into generalized traits in stress hardiness (HARDINESS1 and HARDINESS2 – the first and second principal components, respectively). The opposite of WSD was used (WSD × −1) for PCA so higher values indicated lower hardiness for all three drought traits. Principal components analyses were conducted using the *princomp* function in R (R version 3.0.1; R Core Development Team, Vienna, Austria) and met the assumptions of multivariate normality.

We performed correlation and multiple regressions analyses between PCA scores and seed‐source climate variables to model the effect of climate on stress hardiness. Seed‐source climate variables were calculated with ClimateWNA (Wang et al. 2012). For our regression analyses, PCA scores were modeled using annual and seasonal seed‐source climate variables that have been biologically linked to drought and cold hardiness, with limited autocorrelation (Methods S1 Bansal et al. 2015a,b). Linear models were selected based on AIC_*c*_ scores. The regression equations predicting HARDINESS1 and HARDINESS2 were used to map covariation in stress hardiness across the coastal Douglas‐fir range in ArcGIS v10.1 (ESRI, Redlands, CA).

## Results

**Figure 2:**
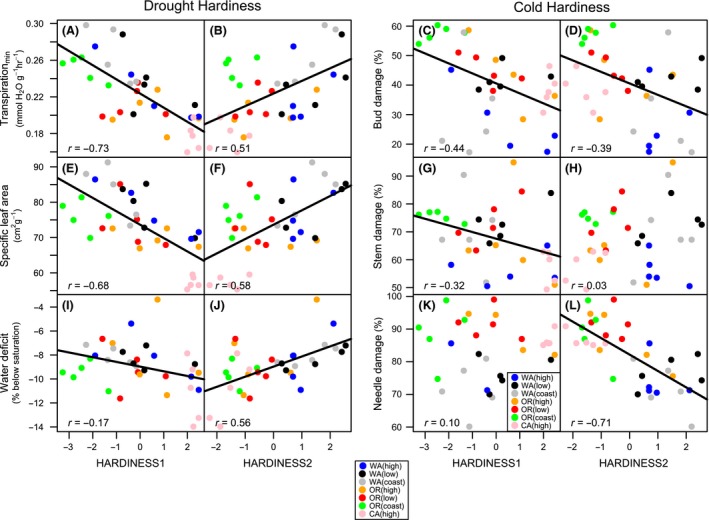
Relationships between the first (HARDINESS1) and second (HARDINESS2) principal component and individual traits associated with drought and cold hardiness for 35 coastal Douglas‐fir (*Pseudotsuga menziesii* var. *menziesii*) populations from seven regions (region colors in legend). Drought hardiness traits include (A, B) transpirationmin, (E, F) specific leaf area, and (I, J) water saturation deficit; cold hardiness traits included (C, D) bud damage, (G, H) stem damage, and (K, L) needle damage. Lines with associated r values are presented only where significant (*P *<* *0.001) correlations existed between principal component scores and individual traits of drought and cold hardiness.

In our principal components analyses, the first (HARDINESS1) and second (HARDINESS2) components explained 47% and 30%, respectively, of the variation among populations in drought and cold hardiness traits. The remaining components explained little variation, and therefore, only the first two components were used for further analyses. Relatively high values of HARDINESS1 were indicative of populations with greater cold hardiness (i.e., less tissue damage in buds and stems) and greater drought hardiness (i.e., lower SLA, transpiration_min_, slightly greater WSD) (Table 1; Fig. 2). Relatively high values of HARDINESS2 indicated greater needle and bud cold hardiness, but there was no significant relationship of HARDINESS2 with stem damage; higher values of HARDINESS2 were indicative of lower drought hardiness (Table 1; Fig. 2).

**Table 1:** Loadings of the first (HARDINESS1) and second (HARDINESS2) principal components on cold and drought hardiness traits measured on 35 coast Douglas‐fir (*Pseudotsuga menziesii* var. *menziesii*) populations

Trait	Stressor	HARDINESS1	HARDINESS2
Bud damage	Cold	−0.541	−0.214
Stem damage	Cold	−0.477	−0.337
Needle damage	Cold	−0.284	−0.561
Transpiration_min_	Drought	−0.438	0.389
Specific leaf area	Drought	−0.411	0.441
Water saturation deficit (× ‐1)	Drought	−0.197	0.424

Values of HARDINESS1 and HARDINESS2 were strongly related to seed‐source climate variables (Table 2). HARDINESS1 was negatively correlated with temperature, particularly with winter minimums, indicating that populations with higher HARDINESS1 values were from locations with relatively cool winters. HARDINESS2 had relatively strong positive relationships with summer precipitation and negative with summer aridity (Table 2), indicating that populations with lower HARDINESS2 values were from locations with warmer, drier summers. Our multiple regression analyses indicated that the best model for predicting HARDINESS1 and HARDINESS2 was as follows:

$$\text{HARDINESS1}=3.02-0.68\times \text{MCMT}\;(R^{2}=0.66)$$

$$\text{HARDINESS2}=-1.97+0.007\times \text{MSP}\;(R^{2}=0.50)$$

**Table 2:** Pearson correlation coefficients for relationships between the first (HARDINESS1) and second (HARDINESS2) principal components and geographic/climate variables associated with the location where seeds were collected for 35 coast Douglas‐fir (*Pseudotsuga menziesii* var. *menziesii*) populations. The traits used for PCA were associated with drought and cold hardiness. Higher values in HARDINESS1 are indicative of greater drought and cold hardiness. Higher values in HARDINESS2 are indicative of greater drought hardiness but lower cold hardiness

Climate	HARDINESS1	HARDINESS2
Elevation	**0.70** ***	−0.37*
Latitude	−0.33	**0.84** ***
Longitude	**0.85** ***	−0.14
Mean annual temp.	−**0.65** ***	−0.34*
Mean coldest month temp.	−**0.82** ***	−0.19
Ending frost free day	−**0.82** ***	−0.17
Precipitation as snow	0.62**	0.25
Extreme minimum temperature	−**0.85** ***	0.47
Mean warmest month temp.	−0.19	−**0.55** ***
Extreme maximum temperature	−0.28	**0.62** ***
Mean annual precipitation	−0.33	0.50**
Mean summer precipitation	−0.14	**0.72** ***
Summer heat‐to‐moisture index	−0.21	−**0.70** ***

Summer heat‐to‐moisture index = (Mean warmest month temp / Mean summer precipitation). Climate variables used for correlations were derived from ClimateWNA. Significance of correlation coefficients: *P <* *0.05, **P <* *0.01, ***P <* *0.001 and in bold.

For HARDINESS1, the negative coefficient for MCMT (mean coldest month temperature) indicates that those populations with lower winter temperatures have higher HARDINESS1 values (i.e., greater drought and cold hardiness). For HARDINESS2, the positive coefficient for MSP (mean summer precipitation) indicates that those populations with lower summer precipitation have lower HARDINESS2 scores (i.e., higher drought hardiness but lower cold hardiness).

**Figure 3:**
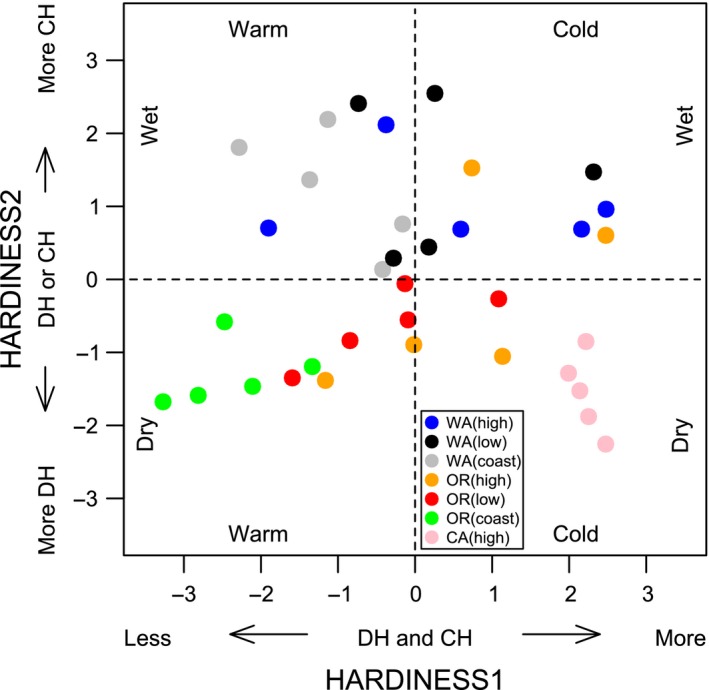
A scatter plot of the first two principal component scores (HARDINESS1 and HARDINESS2) for 35 populations from seven regions (colors in legend) of coastal Douglas‐fir (*Pseudotsuga menziesii* var. *menziesii*). Populations with higher HARDINESS1 scores (right two quadrants) had relatively high drought (DH) and cold (CH) hardiness, and originated in locations with relatively cold winters. Populations with lower HARDINESS2 scores (bottom two quadrants) had relatively high drought hardiness at the expense of cold hardiness, and originated in locations with dry summers.

Populations from southeastern regions of the coastal Douglas‐fir range had relatively high HARDINESS1 and low HARDINESS2 values compared to central and northern populations (Fig. 3), which is associated with cold winters and dry summers of the California Sierra Nevada. Northern populations tended to have similar HARDINESS1 values as central populations, but with relatively high HARDINESS2 values (Fig. 3), indicating the Washington populations were relatively cold hardy while Oregon populations were relatively drought hardy (Fig. 3).

**Figure 4:**
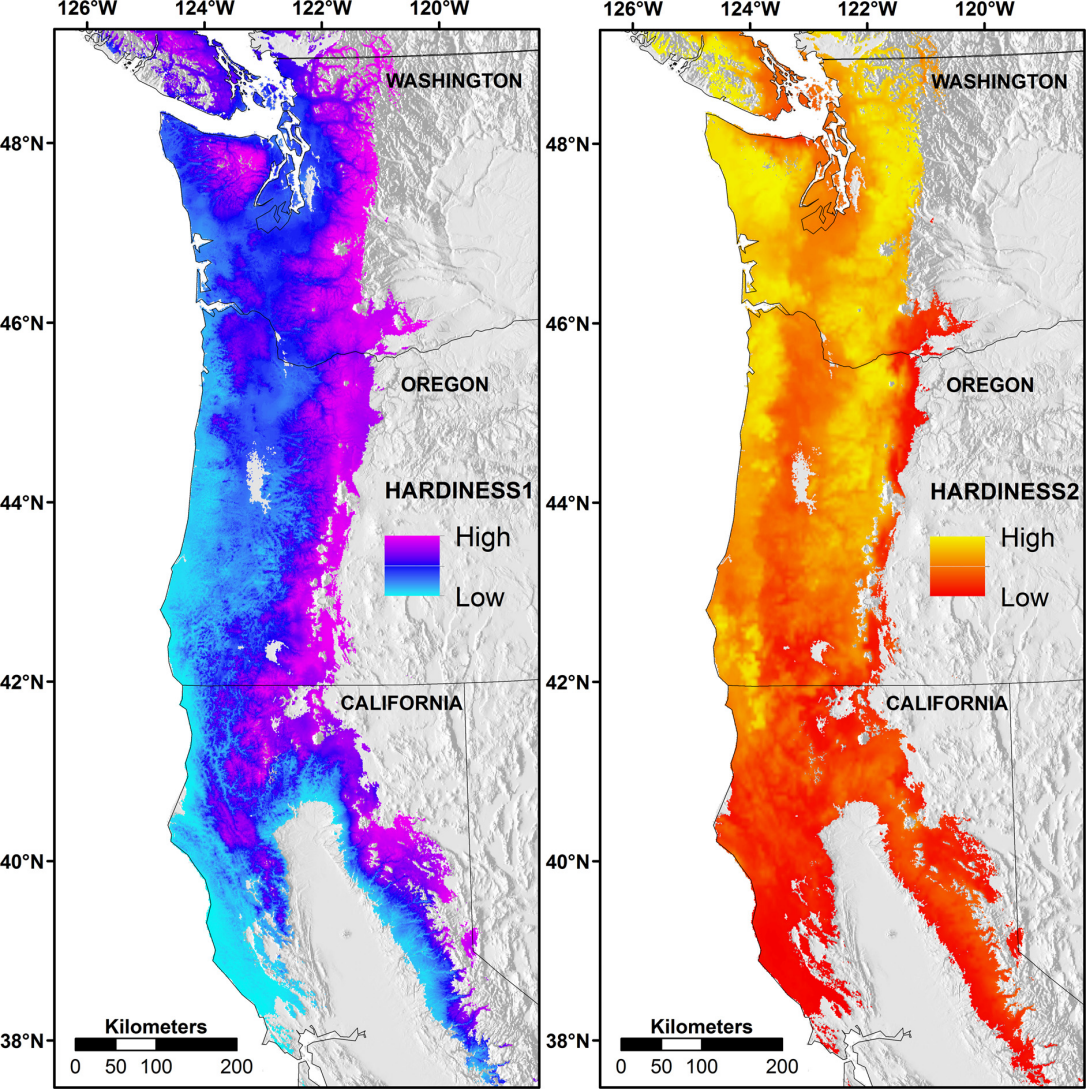
Geographic variation in the first (HARDINESS1) and second (HARDINESS2) principal components of coastal Douglas‐fir (*Pseudotsuga menziesii* var. *menziesii*) in the Pacific Northwest, USA. The principal components were derived by combining drought and cold hardiness trait data from two common gardens. The components were then modeled using seed‐source climate variables. HARDINESS1 values exhibited a strong longitudinal gradient associated with elevation and cool winter temperatures; HARDINESS2 values exhibited a latitudinal gradient associated with summer precipitation. Higher values of HARDINESS1 correspond with greater drought *and* cold hardiness. Higher values of HARDINESS2 correspond with greater cold hardiness *but reduced* drought hardiness.

When HARDINESS1 and HARDINESS2 were modeled across the Douglas‐fir range, predicted HARDINESS1 values exhibited a strong longitudinal gradient associated with elevation and cool temperatures, with relatively high values in the eastern mountainous regions along the Cascade Crest and Sierra Nevada (Fig. 4). Predicted HARDINESS2 values exhibited a latitudinal gradient associated with summer precipitation, with lower values in southern regions (Fig. 4).

## Discussion

Research in the climate change arena has largely focused on the effects of increasing average temperatures, in part because of relatively high confidence in mean temperature prediction models. However, changes in precipitation and in extremes of both temperature and precipitation are primary drivers of natural selection and will have considerable impact on species and population dynamics in the future (VanDerWal et al. 2013; Monleon and Lintz 2015; Renwick and Rocca 2015). We revealed how variation in tolerance to drought and cold stress among Douglas‐fir populations appears influenced by both temperature *and* precipitation gradients.

Drought and cold hardiness converged among populations along winter temperature gradients, meaning that Douglas‐fir populations from cooler climates were inherently more tolerant to both stressors. The reason is likely due to common adaptive mechanisms at the cellular and tissue levels for coping with frost and winter desiccation (White 1987). During a frost event, liquid water in the intercellular spaces in leaves freezes first (Ashworth 2010), creating a water potential gradient between inter‐and intracellular water (Levitt 1980). In addition, sunny days and dry winter winds increase the vapor pressure deficit between plant shoots and the air, while frozen soils limit moisture uptake by the roots (Tranquillini 1982), exacerbating winter desiccation stress. Cellular dehydration induces a number of stress‐tolerance genes and physiological adjustments, such as the accumulation of osmoprotectants (Kasuga et al. 1999), dehydrins (Richard et al. 2000), sugar alcohols (Nakashima et al. 2014), and raffinose oligosaccharides (Taji et al. 2002), which are common mechanisms for coping with winter or summer drought. A reduction in water loss from increased epicuticular wax thickness on needles has also been linked with drought hardiness in both winter (Hadley and Smith 1990) and summer (Bansal et al. 2015a; Harrington and Carlson *in press*). Thus, a degree of tolerance to both drought and cold is likely common across plant taxa that experience periodic subzero temperatures.

Despite strong covariation in drought and cold hardiness among populations along a winter temperature gradient, tolerance to these two stressors diverged along a summer precipitation gradient. Trade‐offs in growth for stress tolerance (Bansal et al. 2015a), or in tolerances to one stressor at the expense of another (Laanisto and Niinemets 2015), are fundamental properties of organismal biology that are often observed across and within taxa (Colautti et al. 2010). Even though cellular dehydration is a common consequence to both drought and cold stress, there may be considerable differences in the initial cues that trigger these stress responses (e.g., osmotic stress vs. cold temperatures), the respective genes and signally pathways that are induced (e.g., ABA dependent vs. ABA independent), physiological adjustments (e.g., stomatal closure vs. membrane restructure), and ultimate causes of cell death (e.g., ionic toxicity vs. physical destruction) (Mahajan and Tuteja 2005). These differences demonstrate how evolutionary pressures and physiological trade‐offs have produced an array of unique stress response networks (Howe et al. 2003; McDowell 2011), which may require specializations that conflict between stressor. Notably, the loss of cold in exchange for drought hardiness was particularly strong for needle, moderate for bud, and absent for stem tissues, suggesting the trade‐off mechanisms may relate to photosynthesis. Evolutionary and applied geneticists seeking to elucidate (and potentially enhance) the molecular mechanisms that confer tolerance to one stressor must therefore consider the linkages to nontarget stressors that may have convergent (but potentially divergent) mechanisms of tolerance (Nakashima et al. 2014).

This study demonstrates how coastal Douglas‐fir has differentiated with respect to drought and cold hardiness along two distinct environmental gradients, leading to a highly structured and predictable geographic distribution of stress hardiness across the species' range. Given the current and future persistence of drought and frost events (I.P.C.C. 2013), tolerance to both stressors must be considered with regard to assisted migration of populations as a management strategy for coping with climate change. Due to the convergence of drought and cold hardiness along *temperature* gradients, populations transferred from warmer to cooler environments may actually have lower drought and cold hardiness than those originating from cooler climates. However, drought hardiness can be increased through selection of populations from warmer *and* drier climates, although this will exacerbate the loss in cold hardiness because of the divergence in drought and cold hardiness along *precipitation* gradients. In reality, convergence and divergence of traits along multiple climate gradients is occurring for suites of traits. Quantifying and modeling covariation among traits can help develop guidelines for choosing populations within regions for assisted migration that have a combination of relatively high growth potential *and* reasonable stress tolerance, thus meeting the goals of assisted migration.

Research on plant tolerance to multiple stressors has almost exclusively been conducted at the biochemical and molecular scales, and is primarily focused on crop species and/or *Arabidopsis thaliana* (Kasuga et al. 1999; Pembleton and Sathish 2014). In many crop species, attempts to overexpress genes or transcription factors to confer tolerance to multiple stressors are currently the subject of intensified research due to concerns over food security with a growing global population (Nakashima et al. 2014). In comparison, similar research on coniferous trees is limited (Jarvis et al. 1996; Richard et al. 2000; Sork et al. 2013; Bansal et al. 2015b). Given the importance of conifers as a fundamental component to many ecosystems, and the potential assisted migration of populations beyond their current ranges, understanding covariation in stress tolerances of conifers is of paramount importance (Neale and Kremer 2011; Alberto et al. 2013). This information is not only useful for modeling and management of species, but also provides a unique opportunity to link upstream molecular mechanisms involved with stress tolerance to downstream ecological impacts. Our study improves our understanding of how multiple climate variables may have influenced the evolution and expression of tolerance to both drought and cold stress of Douglas‐fir, and also demonstrates an experimental and analytical framework for linking multiple climate variables to multivariate traits across many scales.

## Conflict of Interest

None declared.

## Supporting information


**Methods S1**. Combinations of Minimum Cold Month Temperature and Mean Summer Precipitation of the location where seeds for each population were collected.
**Methods S2**. Experimental design.
