# Supplementary material for: Tolerance to multiple climate stressors: a case study of Douglas‐fir drought and cold hardiness
Source: Ecol Evol. 2016 Feb 26;6(7):2074–83. doi: 10.1002/ece3.2007 (PMC4831441; doi:10.1002/ece3.2007)

Supplementary Methods 1

Combinations of Minimum Cold Month Temperature and Mean Summer Precipitation of the location where seeds for each population were collected.


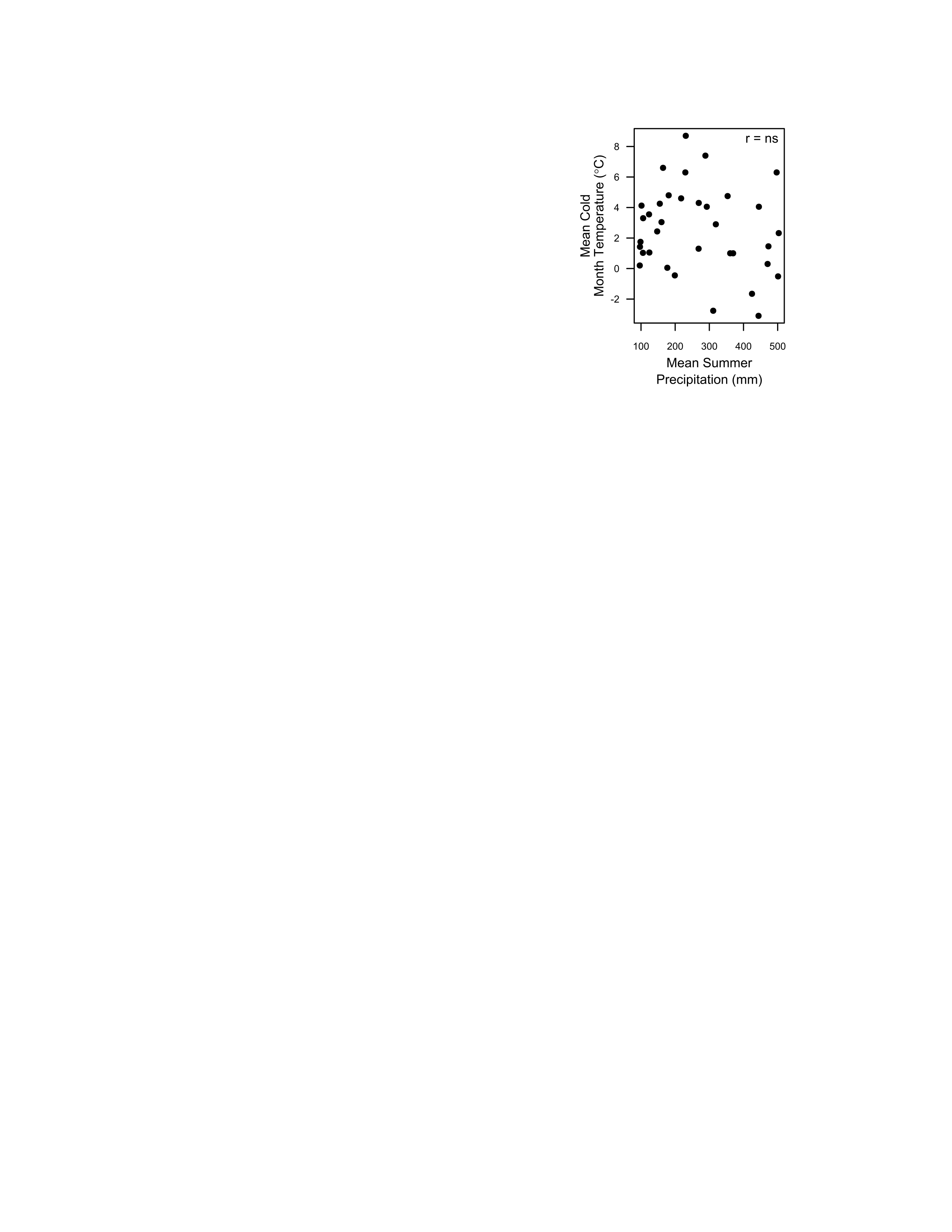


Supplementary Methods 2: Experimental design. Note: Moderate test site not used for current analyses.


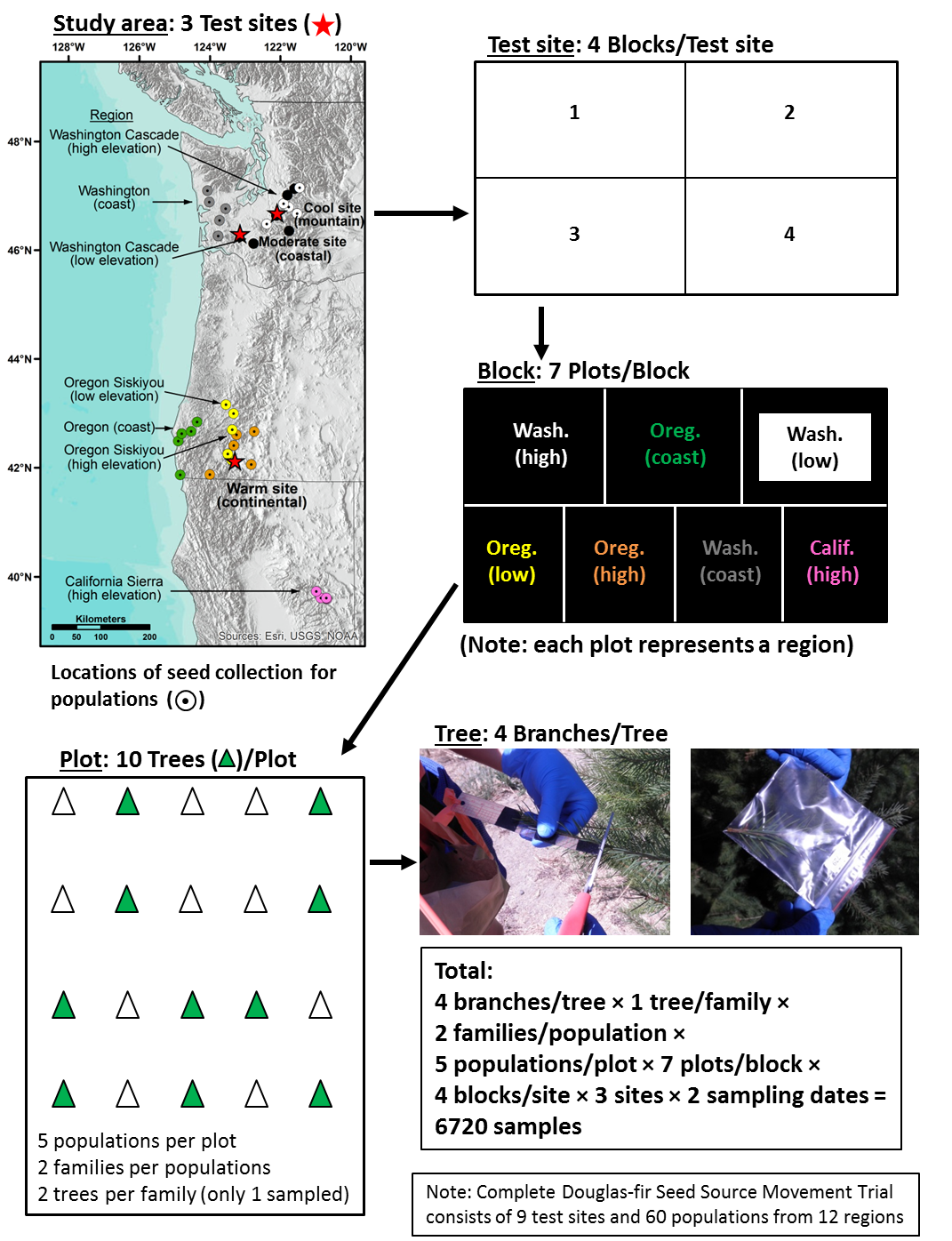

Supplement: Supplementary file 1 — Methods S1. Combinations of Minimum Cold Month Temperature and Mean Summer Precipitation of the location where seeds for each population were collected. Methods S2. Experimental design. [file ECE3-6-2074-s001.docx]
